# Poly(2-Deoxy-2-Methacrylamido-D-Glucose)-Based Complex Conjugates of Colistin, Deferoxamine and Vitamin B12: Synthesis and Biological Evaluation

**DOI:** 10.3390/pharmaceutics16081080

**Published:** 2024-08-17

**Authors:** Mariia Stepanova, Mariia Levit, Tatiana Egorova, Yulia Nashchekina, Tatiana Sall, Elena Demyanova, Ivan Guryanov, Evgenia Korzhikova-Vlakh

**Affiliations:** 1 Institute of Macromolecular Compounds of Russian Academy of Sciences, 199004 St. Petersburg, Russia; maristepanova@hq.macro.ru (M.S.); musia_1@yahoo.com (M.L.); 2 Institute of Highly Pure Biopreparations, 197110 St. Petersburg, Russia; egorova25tat@yandex.ru (T.E.); lenna_22@mail.ru (E.D.); 3 Institute of Cytology of Russian Academy of Sciences, 194064 St. Petersburg, Russia; ulychka@mail.ru; 4 Institute of Experimental Medicine, 197022 St. Petersburg, Russia; miss_taty@mail.ru; 5 Institute of Chemistry, St. Petersburg State University, 198504 St. Petersburg, Russia

**Keywords:** synthetic glycopolymer, covalent conjugates, polymyxins, colistin, siderophore, vitamin B12, deferoxamine, complex conjugates, antimicrobial activity, in vitro permeability assay

## Abstract

Growing resistance to traditional antibiotics poses a global threat to public health. In this regard, modification of known antibiotics, but with limited applications due to side effects, is one of the extremely promising approaches at present. In this study, we proposed the synthesis of novel complex polymeric conjugates of the peptide antibiotic colistin (CT). A biocompatible and water-soluble synthetic glycopolymer, namely, poly(2-deoxy-2-methacrylamido-*D*-glucose) (PMAG), was used as a polymer carrier. In addition to monoconjugates containing CT linked to PMAG by hydrolyzable and stable bonds, a set of complex conjugates also containing the siderophore deferoxamine (DFOA) and vitamin B12 was developed. The structures of the conjugates were confirmed by ^1^H NMR and FTIR-spectroscopy, while the compositions of conjugates were determined by UV–Vis spectrophotometry and HPLC analysis. The buffer media with pH 7.4, corresponding to blood or ileum pH, and 5.2, corresponding to the intestinal pH after ingestion or pH in the focus of inflammation, were used to study the release of CT. The resulting conjugates were examined for cytotoxicity and antimicrobial activity. All conjugates showed less cytotoxicity than free colistin. A Caco-2 cell permeability assay was carried out for complex conjugates to simulate the drug absorption in the intestine. In contrast to free CT, which showed very low permeability through the Caco-2 monolayer, the complex polymeric conjugates of vitamin B12 and CT provided significant transport. The antimicrobial activity of the conjugates depended on the conjugate composition. It was found that conjugates containing CT linked to the polymer by a hydrolyzable bond were found to be more active than conjugates with a non-hydrolyzable bond between CT and PMAG. Conjugates containing DFOA complexed with Fe^3+^ were characterized by enhanced antimicrobial activity against *Pseudomonas aeruginosa* compared to other conjugates.

## 1. Introduction

According to the World Health Organization (WHO), increasing resistance to traditional antibiotics poses a global threat to public health [1]. The search for new antibacterial substances is of great importance and at the same time a long and expensive process. In this regard, modification of known antibiotics, but with limited applications due to side effects, seems to be an extremely promising approach at present [2,3]. These potential candidates include polymyxins, which are cyclic cationic peptides containing an aliphatic tail and are active mainly against Gram-negative pathogens [4,5]. Similar to cationic surfactants, polymyxins disrupt the membrane of bacterial cells by interacting with their anionic phospholipids [6]. This feature of their action leads to the rare development of resistance to polymyxins in susceptible bacteria.

Depending on the amino acid composition, polymyxins A, B, C, D, E (colistin), and M are distinguished. Among them only polymyxin B and colistin are used in the clinic [7]. However, the low stability of polymyxins in the bloodstream requires the administration of high doses of the drug to achieve a therapeutic effect, which, in turn, is accompanied by their nephro- and neurotoxicity. As a result, to date, their use has been limited mainly to topical systems (transdermal, inhalation, or as components of eye and ear drops) [5,8]. In severe infectious processes caused by multidrug-resistant bacterial strains, polymyxins can be used as deep reserve drugs in combination with other antibiotics [6,9].

The existing toxic side effects can be minimized by using different polymyxin delivery systems. The use of a delivery system can reduce the toxicity of polymyxins, prolong their stability, and modify their pharmacokinetics. Currently, various polymyxin delivery systems based on physically loading a drug substance into the delivery system or its covalent conjugation with the polymer carrier have been proposed for parenteral, oral, or topical administration [10,11,12,13,14]. In particular, the preparation of nanoparticles due to interpolyelectrolyte complexation of colistin with hyaluronan [15], hyaluronan/diethylaminoethyl chitosan [16], poly(glutamic acid-*co*-phenylalanine) [17,18], or poly(glutamic acid) followed by further stabilization with 1,2-dimyristoyl-sn-glycero-3-phosphoethanolamine-*N*-(methoxy(poly(ethylene glycol)-2000) [19] have been reported. Moreover, using this approach, combined systems of colistin and tobramycin with oligochitosan/anionic starch for the oral delivery of antibiotics have been developed [20]. Polymyxin B delivery systems are reported to be prepared by complexation with alginate/chitosan [21,22] and poly(glutamic acid)@Ag nanoparticles/agarose hydrogel [23] for oral administration and topical wound treatment, respectively. In addition to the formation of interpolyelectrolyte complexes, drug loading due to hydrophobic interactions is also possible. In this case, lipid nanoparticles [24,25], hydrophobic poly(butyl cyanoacrylate)-based nanoparticles [26], and poly(*L*-lactide)/halloysite nanotube nanofiber mats [27] are reported as delivery systems for polymyxins.

In addition to physically loaded delivery systems, polymer-drug conjugates may be a matter of choice to overcome the known side effects of polymyxins. Nowadays, the synthesis of covalent conjugates of colistin with dextrin [28,29], alginate oligosaccharide [30], poly(ethylene glycol) (PEG) [31], poly(PEG-methyl ether acrylate) [32], and hyaluronan [33], as well as polymyxin B with human immunoglobulin G [34], vancomycin [35], chitosan [36], and poly(glutamic acid-*co*-phenylalanine) or poly(*N*-vinyl succinamic acid-*co*-*N*-vinylsuccinamide) [37] have been reported. The release of the antibiotic from the conjugates occurs by the action of the corresponding enzymes (α-amylase [29,38], esterase or alginate lyase [30], lysozyme [33]) or by non-enzymatic hydrolysis [32,33,37].

The introduction of siderophores into antibiotic delivery systems is one of the most promising approaches to improve antibacterial efficacy through the targeted delivery of antibiotics to bacteria [39]. Siderophores are organic molecules capable of binding iron (III) ions. Iron plays a crucial role in the most important enzymatic processes of many living (micro)organisms, including bacteria. However, bacteria cannot independently transform iron (II) into iron (III) and have to obtain it from the host organism. For this purpose, both Gram-positive and Gram-negative bacteria secrete siderophores into the extracellular environment to capture and import iron in order to maintain their life cycle [40]. The use of siderophores as vectors for targeted delivery of antibiotics to bacteria can be compared to a “Trojan horse” strategy [40,41]. In this case, siderophores help to overcome the membrane-mediated resistance of bacteria that produce various mechanisms of protection. There are a number of studies on the conjugation of a siderophore with a drug that is unable to cross the bacterial membrane [39,40,41,42]. It has been shown that the minimal inhibitory concentration (MIC) of antibiotics with siderophores depends on several factors such as the type of antibiotic, bacterial strain, and the siderophore form (free or complexed with Fe^3+^). Specifically, no improvement in the antimicrobial properties was observed for siderophore conjugates with the peptide antibiotic daptomycin against *Staphylococcus aureus*. At the same time, antimicrobial activity against *Escherichia coli* for some conjugates has been detected [43]. Wencewicz et al. revealed that the same conjugates with siderophores can demonstrate a different antimicrobial activity in the absence and in the presence of complexed Fe^3+^ ions [44]. A fourfold improvement in MIC against *S. aureus* was detected for ciprofloxacin–siderophore conjugates containing complexed Fe^3+^ compared to Fe^3+^-free conjugates.

It is well known that polymyxins have a very low intestinal permeability when administered orally [9]. This obstacle can be overcome by conjugation with vitamin B12 (cyanocobalamin), which has a very high membrane transport in the small intestine [45,46]. This property of vitamin B12 makes it very promising as a targeting ligand to improve the intestinal permeability of drugs [47,48], including peptide molecules [49,50,51]. Recently, Dubashinskaya et al. reported the use of vitamin B12 to increase the intestinal permeability of hyaluronan-colistin conjugates [51]. In vitro experiments on the Caco-2 monolayer showed significantly higher permeability of vitamin B12-modified polymeric colistin conjugates compared to free colistin.

In this study, we propose two targeted strategies for the different administration of CT conjugates as biocompatible sustained-release delivery systems. The first system is based on the conjugation of Fe^3+^-complexed siderophores along with CT to provide targeted delivery of CT to bacterial cells from the blood under intravenous administration. In addition to the components of the first system, the second system also includes conjugated vitamin B12 and was designed for oral targeted delivery of CT. A bioinspired water-soluble synthetic glycopolymer, namely, poly(2-deoxymethacrylamido-*D*-glucose) (PMAG), was selected as the polymeric carrier [52]. PMAG is a nontoxic and biocompatible polymer capable of covalent (bio)conjugation [53]. Deferoxamine (DFOA) was used as a clinically approved [54] model siderophore. The synthesis of monocomponent (CT), two-component (CT and DFOA), and three-component (CT, DFOA, and B12) PMAG-based conjugates was performed in two series differing in the type of bonds (hydrolyzable or non-hydrolyzable) in the conjugate. All conjugates were thoroughly characterized for their structure and composition using a series of physicochemical methods (FTIR, ^1^H NMR spectroscopy, UV–Vis spectrophotometry, and HPLC analysis). CT release from the different conjugates in simulated gastrointestinal fluids was evaluated and compared. Finally, the cytotoxicity of the conjugates in human embryonic kidney (HEK 293) cells, the antimicrobial efficacy of the conjugates against *Pseudomonas aeruginosa*, and the permeability through a Caco-2 monolayer were thoroughly investigated.

## 2. Materials and Methods

### 2.1. Materials

Sodium metaperiodate (≥99%), deferoxamine methanesulfonate salt (≥92.5%), succinic anhydride, *N*,*N*′-diisopropylcarbodiimide (DIC, 99%), 1-hydroxybenzotriazole hydrate (HOBT, ≥97%), and cyanocobalamin (vitamin B12, B12) were purchased from Sigma-Aldrich (Darmstadt, Germany). Polymyxin E (colistin, CT) sulfate salt was the product of Glentham Life Sciences Ltd. (Corsham, UK). 4-(Dimethylamino)pyridine (DMAP, 98%), iron(III) chloride hexahydrate (≥98%), salts for buffer solution preparation, and solvents were supplied by Vecton Ltd. (St. Petersburg, Russia). All solvents were purified by distillation before use. Additionally, dimethyl sulfoxide (DMSO) used for the succinylation of vitamin B12 was dried by 4 Å molecular sieves. 3F powder (also known as ‘SIF Powder’) for the preparation of FaSSGF and FaSSIF biorelevant media simulating gastrointestinal fluids was purchased from Biorelevant (London, UK).

Spectra/Pore dialysis membrane tubes (MWCO 3500) and Amicon^®^ Ultra-4 centrifugal filters (MWCO 3000) were purchased from Repligen Corp. (Boston, MA, USA) and Merck Millipore Ltd. (Carrigtohill, County Cork, Ireland), respectively.

Poly(2-deoxy-2-methacrylamido-*D*-glucose) was synthesized by RAFT polymerization using monomer/RAFT agent/initiator molar ratios of 75:1:0.25 and according to the protocol described elsewhere [55]. The molecular mass characteristics of PMAG were studied by size-exclusion chromatography (SEC) using a Shimadzu HPLC system (Tokyo, Japan) containing a refractometric detector (RID-10A), degasser (FCV-10AL VP), pump (LC-10AD VP), column thermostat (CTO-20A), system controller (SCL-10A VP), and equipped with an Agilent PLgel MIXED-D column (5 µM, 7.5 × 300 mm, Agilent Techn., Santa-Clara, CA, USA). The chromatographic conditions were as follows: eluent, 0.1 M LiBr in *N*,*N*-dimethylformamide; temperature, 50 °C; flow rate, 1 mL/min; and standards, poly(methyl methacrylates) with a molecular weight of 4800–46,900 (Agilent Techn., Santa Clara, CA, USA). The average molecular weight and dispersity of the obtained PMAG were 24,100 and 1.2, respectively.

Solutions for chromatographic analysis were filtered using MCE 0.45 μm (HPLC) and PTFE 0.45 μm (SEC) syringe filters with a diameter of 13 mm (Nantong FilterBio Membrane Co., Ltd., Nantong, China).

### 2.2. Methods

#### 2.2.1. Synthesis of Conjugates

The conjugation of DFOA and CT was carried out by reacting their amino groups with aldehyde groups pre-generated in PMAG. For this purpose, part of the glucose units of PMAG were subjected to oxidation with NaIO_4_ according to a previously developed protocol [52] in deionized water at 4–6 °C in the dark for 24 h. The amount of oxidizing agent was varied to generate different contents of aldehyde groups in PMAG. Specifically, molar ratios of [NaIO_4_]:[MAG] of 0.3:1 and 0.45:1 were used to produce 10 and 20 mol% CHO groups in PMAG [52], respectively. The resulting product was purified by dialysis against water using a dialysis bag (MWCO 3500). On the first day, dialysis was performed at 22 °C with the water changed 6 times, and on the following two days, dialysis was performed in a refrigerator with the water changed 2 times per day.

In the case of DFOA and CT conjugation, a freshly synthesized and freshly purified concentrated aqueous solution of oxidized PMAG (ox-PMAG) was dialyzed against borate buffer (BB, 0.0125 M, pH 9.2) for 1 day. Dialysis was performed for the first 4 h at 22 °C with a change of borate buffer every hour, and the rest of the time, the dialysis was carried out in the refrigerator. Conjugation was performed according to the method described elsewhere [37] to obtain mono- and complex conjugates. To synthesize monoconjugates (PMAG-DFOA or PMAG-CT), DFOA or CT, also dissolved in BB, was added to the ox-PMAG solution in BB (obtained after dialysis). In the case of complex conjugates (PMAG-(DFOA, CT)), a BB solution with both DFOA and CT was added to the ox-PMAG solution in BB.

For monoconjugates, the molar ratios of DFOA/CT to –CHO groups of ox-PMAG were 5:1:1. For complex conjugates, the molar ratios of DFOA:CT:–CHO groups of ox-PMAG were 5:1:1. The reaction was carried out under stirring with a magnetic stirrer at 22 °C for 2 h. The resulting products were purified by dialysis using a dialysis bag (MWCO 3500) against water for 1 day in the refrigerator. After purification, one-half of the solution was withdrawn, and the formed aldimine bonds (Schiff base) and the potentially remaining unreacted aldehyde groups were reduced with sodium borohydride. The reduction was carried out in aqueous medium at 22 °C for 1 h under constant stirring using a 3-fold molar excess of NaBH_4_ with respect to the –CHO groups. Dialysis of the remaining portion of the unreduced conjugates was continued for another 4 days in the refrigerator. At the same time, the conjugates after reduction were also purified by dialysis against water using dialysis bags (MWCO 3500) at room temperature for 4 days. The resulting combined conjugates were freeze-dried. When 800 mg of PMAG was initially used for the synthesis, the yields of the conjugates were 62–67%.

All dialysis washes after conjugation were collected, concentrated using a rotary evaporator, combined, and freeze-dried for subsequent HPLC analysis of the amounts of unreacted DFOA and CT (Section 2.2.2).

Subsequent modification with vitamin B12 of complex conjugates was performed using succinylated vitamin B12 (Suc-B12) previously obtained as described elsewhere [51]. Briefly, Suc-B12 synthesis was carried out in dry DMSO in the presence of an equimolar amount of DMAP at 30 °C for 1 day using a 100-fold molar excess of succinic anhydride. Then, 45 mg (0.031 mmol) of Suc-B12 was dissolved in 3 mL of dry DMSO at 40 °C with stirring on a magnetic stirrer. The resulting solution was cooled to room temperature, and 5.4 μL (0.034 mmol) of DIC and 8.4 mg (0.062 mmol) of HOBT were added to activate the carboxyl groups of Suc-B12 for subsequent interaction with the amino groups of colistin. This reaction was carried out for 40 min at 22 °C. After activation of the -COOH groups, the Suc-B12 solution was transferred to a flask containing 22 mL of dry DMSO and complex conjugate in such an amount that the colistin content was equimolar to Suc-B12. The reaction was carried out at 37 °C and 121 rpm for 4.5 h using an orbital thermoshaker (Unimax 1010, Heidolph, Schwabach, Germany). The complex conjugates with both unreduced and reduced aldimine bonds were used for this modification. The resulting product was purified by dialysis in a refrigerator for 1 week using a dialysis bag (MWCO 3500) and lyophilized. In the case of conjugates with reduced aldimine bonds, dialysis was performed against water, whereas for conjugates with Schiff bases, dialysis was performed against phosphate buffer (0.00125 M, pH 7.4) for the first 6 days and then against water for 1 day to avoid disruption of the aldimine bonds. The yields of the complex PMAG-(DFOA, CT-B12) conjugates were 69–72%.

#### 2.2.2. Characterization of Precursors and Conjugates

Fourier-transform infrared spectroscopy (FTIR) was carried out using an IRAffinity-1 S spectrometer (Shimadzu, Tokyo, Japan) in transmission mode. The FTIR spectra were recorded in the region of 500 to 3500 cm^−1^ at a resolution of 2 cm^−1^ for 40 scans on KBr pellets.

^1^H NMR and ^1^H NMR one-dimensional diffusion ordered (1D-DOSY) spectroscopy was carried out using D_2_O as the solvent at 25 °C. The analysis was performed using an AVANCE AV-400 spectrometer (Bruker, Karlsruhe, Germany).

The amount of conjugated CT and DFOA was calculated as the difference between the initial and unreacted amounts of components. The latter were determined by quantitative HPLC analysis of the samples that were collected, concentrated, and freeze-dried after rigorous purification of the conjugates. HPLC analysis was carried out by ion-exchange HPLC with the use of a Prominence LC-20AD HPLC system (Shimadzu, Kyoto, Japan) with a diode array detector that was equipped with a monolithic analytical CIM SO_3_ disk (5 × 5 mm, Sartorius, Gottingen, Germany) in gradient elution mode. Sodium-phosphate buffer solution (0.005 M, pH 7.0) and 2 M aqueous sodium chloride solution were used as phases A and B, respectively. The tested samples were dissolved in phase A. The analysis conditions were set as follows: 0–2 min, 100% phase A; 2–7 min, 0–100% phase B; 7–15 min, 100% phase B; mobile phase flow rate 0.5 mL/min; detection wavelength 215 nm; and injection volume 20 µL. The retention time of DFOA was 0.89 (main peak) and 5.4–5.7 (minor peak). The retention time of CT was 8.8–9.2 min. Quantitative determination was carried out using calibration plots for DFOA and CT built in the range of concentrations of 0.05–0.75 and 0.005–5.00 mg/mL, respectively.

The efficiency of vitamin B12 conjugation was determined by UV–Vis spectrophotometry using an SF-56 spectrophotometer (SPECTR LLC, St. Petersburg, Russia). The quantitative analysis of vitamin B12 contained in the conjugates was carried out at a wavelength of 550 nm using a calibration plot built for vitamin B12 in the concentration range of 0.003–0.100 mg/mL.

The conjugation efficacy (*CE*, %) of DFOA, CT, or vitamin B12 was determined using the following equation:

(1) $$CE\;(\%)=\frac{m_{C}}{m_{0}}*100$$

where *m_C_* is the mass of the conjugated substance (CT, DFOA, or vitamin B12), *m*_0_ is the initial mass of the substance taken for the reaction. In the case of DFOA, an amount equimolar to the aldehyde groups was used as the theoretically possible *m*_0_. In fact, it is equal to the mass of DFOA taken for the reaction divided by 5.

The purity of the conjugates was determined by HPLC. With this aim, phase A was added to the weighed purified conjugate sample. The freshly prepared system was centrifuged using an Amicon^®^ Ultra-4 membrane tube at 20 °C, 4215× *g* for 10 min. The ultrafiltration was repeated three times. The amounts of free CT and DFOA in the collected fractions were analyzed by HPLC. The HPLC conditions were the same as described above for the determination of CT and DFOA in the washes. In the case of vitamin B12-containing conjugates, sample preparation was carried out in the same way, but the amount of unreacted Suc-B12 was determined spectrophotometrically as described in the same section above.

#### 2.2.3. Antibacterial Activity Study

The powders of all substances were sterilized by UV for 40 min before the experiment. To obtain iron(III)-complexed conjugates, 3 mg of DFOA-containing conjugates was mixed with 1 mL of sterile water after UV sterilization and an aliquot of a syringe filter-sterilized (PES, 0. 22 μm, 13 mm diameter, Jet Biofil, Guangzhou, China) aqueous FeCl_3_ solution (2.5 mg/mL) in a 2-fold molar excess of iron(III) chloride relative to the molar content of DFOA in the conjugate. This system was incubated for about 1 h at 22 °C and then washed several times with water to remove unbound FeCl_3_ using an Amicon^®^ Ultra-4 (MWCO 3000) by centrifugation at 20 °C, 4215× *g*, 10 min. Conjugates of CT with concentrations of 0.25–64 µg/mL (according to CT) were tested to determine the minimum inhibitory concentration (MIC).

The antimicrobial activity of conjugates was examined using the microtiter broth dilution method as previously reported [23,37] using *P. aeruginosa* (ATCC 27853) received from the Museum of Microbiological Cultures of State Research Institute of Highly Pure Biopreparations (St. Petersburg, Russia). Briefly, CT conjugate stock solutions were prepared by diluting samples in Mueller–Hinton broth to a maximum 2-fold concentration equivalent to CT. *P. aeruginosa* inoculum was serially diluted 1:100 in Müller–Hinton broth to obtain a concentration of 10^7^ CFU/mL. For the MIC determination, sterile 96-well flat-bottom plates were used, and 125 µL of *P. aeruginosa* suspension in Mueller–Hinton broth was added to the wells. Then 2× concentrations of CT (125 µL) were added to the wells of the 1st column, resuspended, and transferred to the next column, repeating the antibiotic dilutions until the last column on the plate. In this way, successive dilutions of the antibiotic were obtained at concentrations ranging from 64 µg/mL to 0.25 µg/mL. In addition, sterile Mueller–Hinton broth and non-treated culture growth were used as negative and positive controls. After an 18 h incubation of the plate at 37 °C, the optical density in the wells was measured at 630 nm using an ELx808™ Absorbance Microplate Reader (BioTek, Winooski, VT, USA). The relative bacterial growth (%) was calculated as the ratio of the optical density of the test sample at each concentration to the optical density in the positive control.

#### 2.2.4. Cytotoxicity Study

Human embryonic kidney cells (HEK 293) purchased from the Cell Culture Collection of the Institute of Cytology RAS (St. Petersburg, Russia) were used to study the cytotoxicity of the conjugates. HEK 293 cells were cultured at 37 °C in a humidified atmosphere containing 5% CO_2_ in modified Eagle’s nutrient medium (MEM, Gibco, Billings, MT, USA) containing 1% essential amino acids, 1% *L*-glutamine, 50 μg/mL streptomycin, 50 U/mL penicillin, and 10 vol% thermally inactivated fetal bovine serum (FBS, HyClone, Logan, UT, USA). A total of 10^4^ cells/100 µL/well was seeded into an adhesive 96-well plate and cultured for 1 day. After that, the medium was removed, and 100 µL of MEM with various concentrations of the test conjugates (4–1000 μg/mL with respect to the CT content) was added to each well with cells and incubated for 72 h. At the end of the incubation period, the medium was removed, and 50 μL/well of MEM with 3-(4,5-dimethylthiazol-2-yl)-2,5-diphenyltetrazolium bromide (0.1 mg/mL, MTT, Sigma-Aldrich, St. Louis, MO, USA) was added. The cells were incubated in a CO_2_ incubator for 2 h at 37 °C. After the removal of the supernatant, formazan crystals produced by the metabolically viable cells were dissolved in DMSO (50 μL/well) and transferred to clean wells, followed by measuring the absorbance of the solutions at 570 nm using a multiwell plate reader (ThermoFisher Multiskan Labsystems, Waltham, MA, USA).

#### 2.2.5. Caco-2 Cell Permeability Assay

The human colon adenocarcinoma cells (Caco-2) obtained from the Cell Culture Collection of the Institute of Cytology RAS (St. Petersburg, Russia) were used to study cell permeability. The study was carried out as described elsewhere [51]. Briefly, Caco-2 cells were cultured in Dulbecco’s modified Eagle medium (DMEM, Biolot, St. Petersburg, Russia) containing *L*-glutamine, streptomycin/penicillin and 10% FBS in cell culture flasks (area 75 cm^2^, Jet Biofil, Guangzhou, China) at 37 °C in a CO_2_ incubator under humidified conditions. The cultured cells were re-seeded into a new cell culture flask (1:6 ratio) when the cells reached 80–90% confluence. Then, 10^5^ cells/500 µL/well were seeded onto the apical side of the membrane (1 μm pore diameter, Corning Inc., Corning, NY, USA) in cell culture inserts for 24-well plates. One milliliter of DMEM was added to the basolateral chamber. The medium in the apical and basolateral chambers was changed every other day. The cells formed a confluent monolayer during 7 days and then polarized within 14 days, acquiring the properties of enterocytes (the formation of tight junctions and microvilli on the apical surface of the cells). After 21 days of cultivation, the medium was removed and the monolayer of cells was washed 3 times with PBS (Biolot, St. Petersburg, Russia). The test conjugates in a 0.01 M phosphate buffer solution (pH 7.2) with a concentration of 0.5 mg/mL according to the content of CT, as well as free vitamin B12 and CT as controls taken at the same concentration, were added to the apical chamber in a volume of 0.5 mL. One milliliter of PBS was placed in the basolateral chamber. Every 30 min for 2 h, 1 mL of PBS from the basolateral chamber was replaced by 1 mL of fresh PBS. The vitamin B12 content and the content of B12-containing conjugates equivalent to vitamin B12 were determined spectrophotometrically, and the CT transferred to the lower chamber was determined by HPLC analysis as described in Section 2.2.2.

The *P_app_* was calculated as follows [56]:

(2) $$P_{app}=\frac{dQ}{dt}*\frac{1}{A*C_{0}}$$

where *P_app_* is the apparent permeability coefficient (cm/s), $\frac{dQ}{dt}$ is the permeation rate (µg/s), *A* is the area of the monolayer (0.3 sm^2^), and *C*_0_ is the concentration of the test substance in the apical chamber at the initial time point (µg/mL).

#### 2.2.6. Stability of Conjugates and Colistin Release Study

The stability of conjugates was studied at different pH values using biorelevant media simulating fasted gastric fluid (FaSSGF, pH = 1.6) and fasted intestinal fluid (FaSSIF, pH 6.5), as well as in 0.1 M HCl (pH = 1, gastric medium) and 0.01 M phosphate buffer (pH = 6.8, duodenal medium) [51]. The FaSSGF and FaSSIF solutions were prepared from commercial 3F powder in accordance with the manufacturer’s protocol.

The CT release from conjugates was assessed using PBS (0.01 M, pH = 7.4) and citrate-phosphate buffer (pH = 5.2) mimicking blood pH and the pH of the ileum or the inflamed area [37,51], respectively.

In both cases, 2 mL of the respective medium was added to 2 mg of conjugate and incubated on an orbital shaker at 37 °C and 121 rpm. For stability studies, the conjugates were incubated for 2 h. After that, a part of the solution with released CT was separated from the conjugate using Amicon^®^ Ultra-4 by centrifugation at 20 °C, 4215× *g* for 10 min, and the amount of released CT was determined. In the release assay, 600 µL of the solution was separated at the predetermined time intervals for the analysis of the released CT using Amicon^®^ Ultra-4, and the taken volume was refilled with a fresh portion of the corresponding medium. The CT content in the test solutions was determined by quantitative HPLC analysis as described in Section 2.2.2.

#### 2.2.7. Statistical Analysis

In the case of the CT release and conjugate stability experiments, each assay was performed in three repeats. The MTT assay was carried out in triplicates in two independent series (*n* = 6). The cell permeability assay and antimicrobial activity were analyzed with *n* = 3 and 4, respectively. The row data were processed using Excel MS Office 2019 software (Microsoft, Redmond, WA, USA). All data are presented as the mean value ± SD.

## 3. Results and Discussion

### 3.1. Synthesis and Characterization of PMAG-Based Conjugates

**Figure 1:**
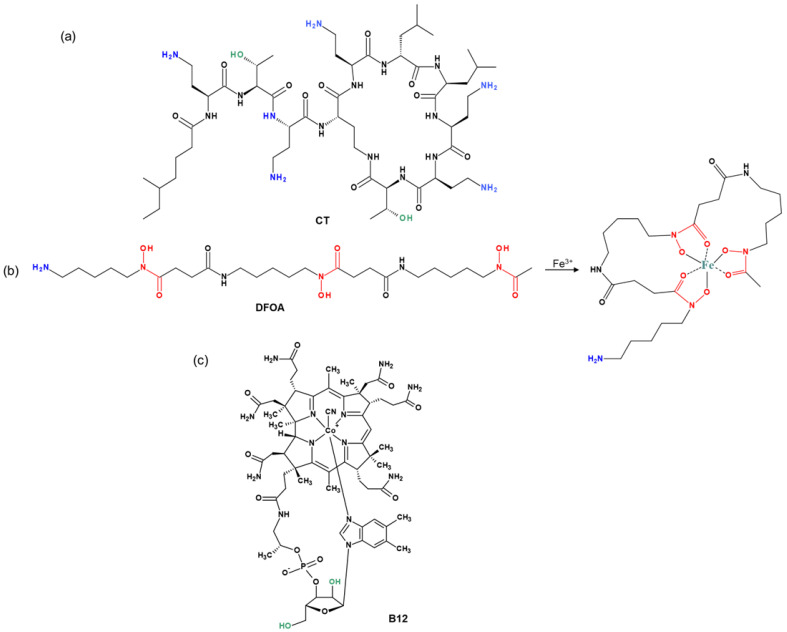
Structures of (**a**) colistin (CT), (**b**) deferoxamine (DFOA), and (**c**) cyanocobalamin (vitamin B12) used for conjugation with PMAG.

The chemical structures of the cyclic peptide antibiotic colistin (CT), the siderophore deferoxamine (DFOA), and vitamin B12 (B12) used for conjugation to PMAG are shown in Figure 1. All components are hydrophilic water-soluble compounds. In addition, the PMAG selected for conjugate synthesis is a neutral polymer. Thus, the occurrence of ionic or hydrophobic interactions, which play a key role in the formation of strong noncovalent complexes between the polymer and other components, is minimized.

**Figure 2:**
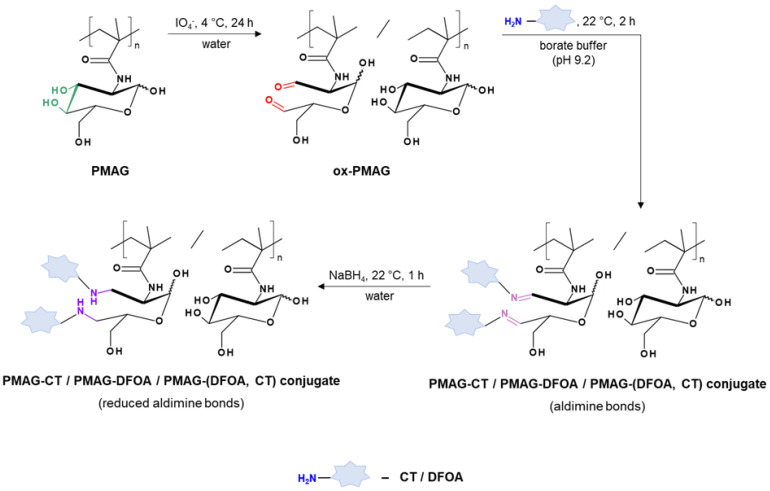
Scheme of modification of PMAG with CT and DFOA to produce mono- (PMAG-CT or PMAG-DFOA) and complex (PMAG-(DFOA,CT)) conjugates.

**Figure 3:**
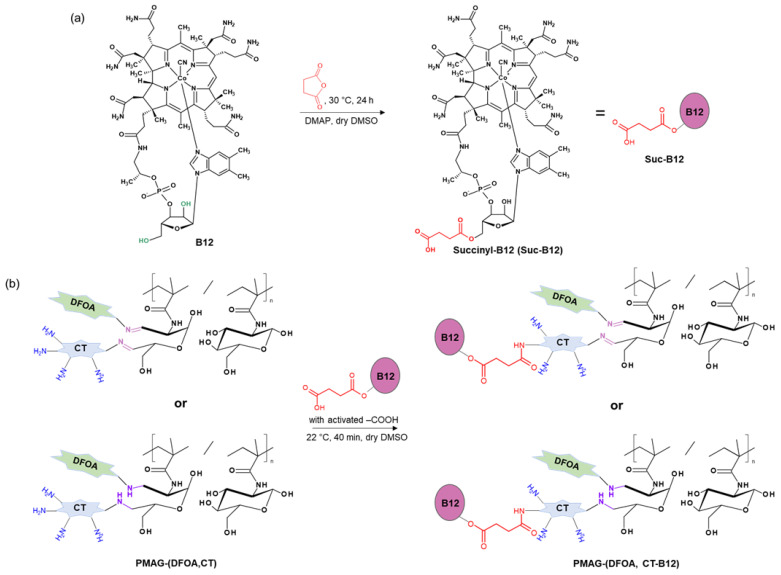
Schemes of (**a**) vitamin B12 succinylation and (**b**) modification of PMAG-based complex conjugates with activated Suc-B12.

Figure 2 shows the scheme of the PMAG modification with CT and DFOA, which is based on the interaction of aldehyde groups pre-generated in PMAG with the amino groups of CT and/or DFOA. Modification of the synthesized complex PMAG(10)-(DFOA,CT) conjugates containing non-reduced and reduced aldimine bonds with vitamin B12 was carried out by reaction of the amino group of CT and pre-activated carboxyl group of succinylated vitamin B12. Schemes of these reactions are illustrated in Figure 3.

The synthesized conjugates were investigated by ^1^H NMR and FTIR spectroscopy in comparison with the neat substances. The ^1^H NMR spectra of the obtained conjugates and initial compounds showed the appearance of signals corresponding to both DFOA and CT (Figure S1, Supplementary Materials). Since most of the signals in the spectra overlapped with the signals of PMAG in monoconjugates or with the signals of both PMAG and co-components in complex conjugates, quantitative calculations of the conjugate composition using this method were not possible. Nevertheless, in the spectra of CT-containing conjugates, signals of the -CH_3_, -CH_2_-, and -CH- groups (0.7–1.3 ppm) of colistin [57,58,59], which partially overlapped with signals of the -CH_3_ groups of PMAG (0.8–1.4 ppm) [55], could be clearly distinguished. Similarly, the spectra of DFOA-containing conjugates showed overlapping signals in the range of 2.4–2.7 and 3.5–3.6 ppm of the -CH_2_- groups of DFOA [60] with signals of the -CH_2_- and -CH- groups of PMAG and CT [55,58] (Figure S1a–c,e, Supplementary Materials). As a result of the modification of the conjugates with vitamin B12, the ^1^H NMR spectra of these conjugates showed, in addition to the indicated DFOA and CT signals, the appearance of a number of signals at 1.37, 1.83, 2.23, and 2.5–2.7 ppm, as well as an increase in the intensity of signals around 1.2 ppm corresponding to the -CH_3_ and -CH_2_- groups of B12 compared to the 0.8 ppm signal for the conjugate PMAG-(DFOA,CT) (Figure S1d–f, Supplementary Materials). However, these signals also partly overlapped with the CT, DFOA, and PMAG signals, also providing only qualitative confirmation of the conjugation. The broadening of the CT, DFOA, and vitamin B12 signals after conjugation with PMAG, which is typical for polymeric products, is also an indicator of the presence of these components in the polymeric formulation.

**Figure 4:**
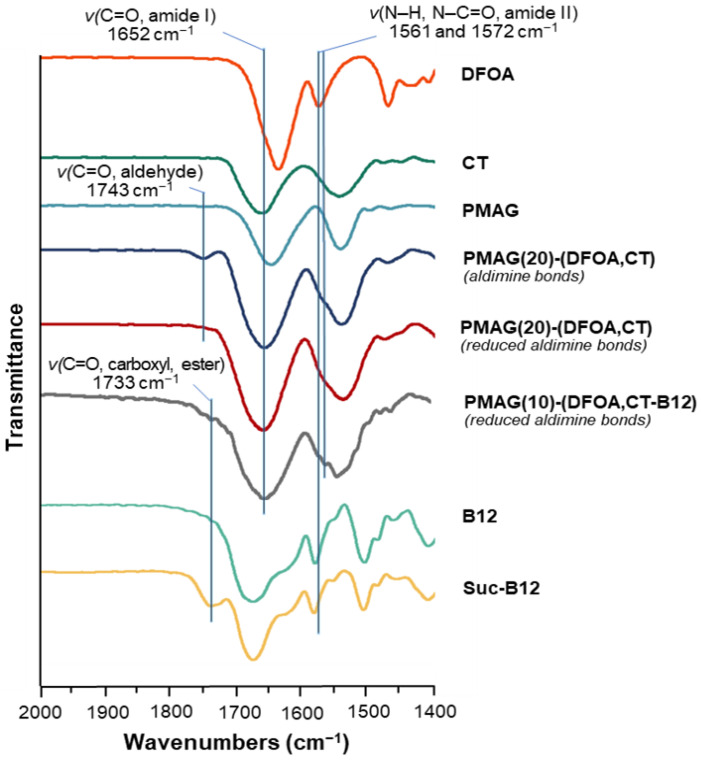
Fragment of FTIR spectra of PMAG-based conjugates and neat substances.

In the FTIR spectra of the CT-containing PMAG-based conjugates, a shift in the characteristic band corresponding to the stretching vibrations of C=O (Amide I) of CT to 1652 cm^−1^ [61] is detected (Figure 4 and Figure S3 of Supplementary Materials). This is in contrast to unmodified PMAG for which the maximum is at 1640 cm^−1^. The FTIR spectra of conjugates with aldimine bonds showed the presence of a characteristic band at 1743 cm^−1^, which disappeared after the reduction of the conjugate, also containing residual aldehyde groups, with sodium borohydride. Hence, this characteristic band can be attributed to the stretching C=O vibrations of the aldehyde groups [62]. The FTIR spectra of succinylated vitamin B12, as well as the FTIR spectra of Suc-B12-containing complex conjugates, demonstrated the presence of a characteristic band at 1733 cm^−1^ corresponding to the stretching C=O vibrations of carboxyl and ester groups [51,63]. Furthermore, the FTIR spectra of DFOA- and vitamin B12-containing conjugates showed the appearance of a shoulder or a band at 1561 and 1572 cm^−1^, which can be attributed to the vibrations of Amide II (deformation N–H and stretching N–C=O vibrations) [63] of DFOA and vitamin B12, respectively. Overall, based on the results of ^1^H NMR and FTIR spectroscopy, it can be concluded that the covalent conjugation of CT, DFOA, and Suc-B12 with PMAG was successful.

In addition, Figure S2 (Supplementary Materials) shows photographs of the initial PMAG and the purified complex conjugates before and after B12 modification. As a result of PMAG (white powder) modification, the color of the products changed to yellow for the complex conjugate and crimson red for the B12-modified complex conjugate, which also indirectly indicates that the modification occurred successfully.

The results of the conjugation of CT and DFOA depending on the content of aldehyde groups in oxidized PMAG are presented in Table 1. In order to synthesize monoconjugates, only CT or DFOA was conjugated with ox-PMAG. Since CT has five free amino groups suitable for conjugation and DFOA has only one, the latter was used in five-fold excess relative to the aldehyde groups, while CT was used in equimolar content relative to the aldehyde groups. Using such ratios, mono- and complex conjugates with an efficacy of conjugation of 41–52% for CT and 41–57% for DFOA were obtained. In the complex conjugates, the efficacy of conjugation of aldehyde groups reached almost 100%, of which the efficacy of conjugation for CT and DFOA was close to 50%. Moreover, the amounts of CT conjugated to 1 mg of ox-PMAG were very close in the synthesis of both the mono- and complex conjugates. This result may be due to the presence of steric hindrance in the reaction of a large amine-containing ligand, such as CT, with the polymer, appearing already at the monoconjugate level.

The results for the modification of PMAG(10)-(DFOA,CT) conjugates containing non-reduced and reduced aldimine bonds with Suc-B12 are summarized in Table 2. It can be seen that the modification of PMAG(10)-(DFOA,CT) conjugates with B12 using the selected strategy was successful and that the efficacy of conjugation was 97–98% for both conjugates.

The conjugates used in further experiments and the contents of components per mg of conjugate are summarized in Table 3. The contents of CT and DFOA in the complex conjugates ranged from 180 to 415 μg CT/mg of conjugate and from 170 to 325 μg DFOA/mg of conjugate, respectively (Table 3). The results obtained for CT appeared to be close to the recently published data on the conjugation of CT with hyaluronic acid by activation of its carboxyl groups followed by interaction of the formed activated esters with amino groups of CT [33]. The reported contents of CT in conjugates with hyaluronic acid were 129–377 μg/mg.

### 3.2. In Vitro Biological Properties of Conjugates

#### 3.2.1. Antibacterial Activity

**Figure 5:**
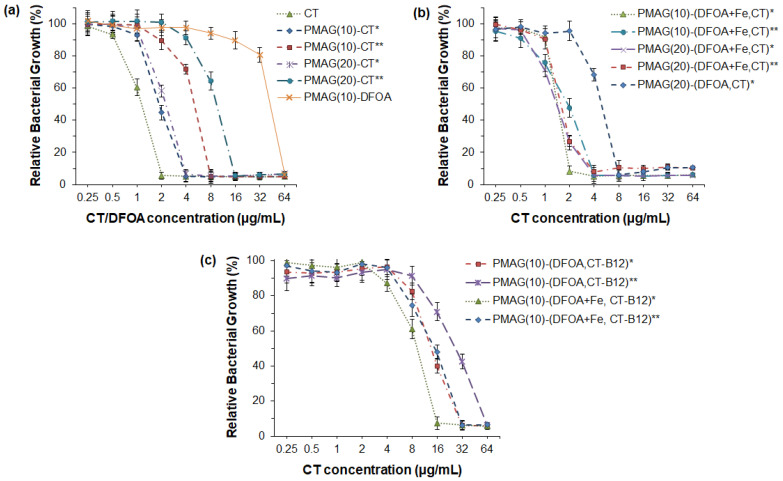
Antibacterial activity of CT and its various PMAG-based conjugates against *P. aeruginosa* (18 h): (**a**) monoconjugates of CT or DFOA, (**b**) double conjugates containing both CT and DFOA; (**c**) three-component conjugates containing CT, DFOA and vitamin B12. * Non-reduced aldimine bonds; ** reduced aldimine bonds.

The antibacterial activity of free CT and its polymeric conjugates was examined against *P. aeruginosa*. Neither DFOA nor PMAG is known to possess antibacterial properties against *P. aeruginosa* in the concentration range of 0.25–64 µg/mL [37]. At the same time, all CT conjugates expectedly showed antimicrobial activity (Figure 5). When analyzing the results of the MIC values for the conjugates studied, the following trends can be distinguished. The best antibacterial activity (MIC = 4 μg/mL) was exhibited by monoconjugates (PMAG(10)-CT and PMAG(20)-CT), in which CT was linked to the polymer through aldimine bonds (Figure 5a). The activity of these conjugates was very close to that of free CT (MIC = 2 μg/mL). A reduction of the aldimine bonds in the conjugates was accompanied by a decrease in the MIC values of monoconjugates to 8 μg/mL for PMAG(10)-CT and 16 μg/mL for PMAG(20)-CT. This decrease may be attributed to a slower rate of interaction of the polymeric form of the antibiotic with the bacterial cell because of the decreased diffusion of conjugates and steric limitations. In addition, this may also be a result of a slower rate of drug release due to the reduction of aldimine bonds, leading to the formation of more stable bonds between CT and PMAG. The higher MIC for PMAG(20)-CT compared to PMAG(10)-CT can be explained by the large number of aldehyde groups formed in PMAG, which leads to pronounced cross-linking with CT, consuming CT amino groups, while the latter affects the activity of the antibiotic.

As mentioned in the introduction, the use of siderophores can improve the delivery of antibiotics, since siderophore-mediated transport of Fe^3+^ ions to bacteria is a natural mechanism to maintain their life cycle. Recently, PMX B-polymer conjugates containing DFOA without Fe^3+^ were shown not to improve the antibacterial properties [37]. In turn, Wencewicz et al. reported that the same conjugates bearing siderophores without and complexed with Fe^3+^ ions exhibit a different behavior [44]. In particular, a series of conjugates of loracarbef and ciprofloxacin with siderophores was synthesized, and their antibacterial activity was investigated. The strongest activity for both conjugated and free antibiotics was detected against *S. aureus*. Moreover, a four-fold improvement in MIC was revealed for ciprofloxacin–siderophore conjugates in the presence of Fe^3+^ ions.

In this study, a set of PMAG-based complex conjugates containing CT and DFOA in complexes with Fe^3+^ were tested for their antibacterial activity, which was compared with the activity of the complex conjugate without Fe^3+^ ions (Figure 5b). The lowest MIC (2 μg/mL) comparable with free CT was detected for the PMAG(10)-(DFOA+Fe,CT) conjugate with aldimine bonds between CT/DFOA and the polymer. As for the monoconjugates, the MIC value for the complex conjugate, but with reduced aldimine bonds, was higher (4 μg/mL). Thus, in both cases, the MIC values for PMAG(10)-(DFOA+Fe,CT) complex conjugates containing DFOA+Fe^3+^ were lower than in the case of the monoconjugate PMAG(10)-CT. A similar tendency was observed for a series of PMAG(20)-(DFOA+Fe,CT). In this case, for both conjugates with reduced and non-reduced aldimine bonds, the MIC values were 4 μg/mL. The four-fold decrease in the MIC for PMAG(20)-(DFOA+Fe,CT)** compared to PMAG(20)-CT** can be a result of the improved interaction of the conjugate with bacteria mediated by the DFOA-Fe^3+^ complex. The antibacterial activity of the PMAG(20)-(DFOA,CT) complex conjugate with aldimine bonds in the absence of Fe^3+^ was determined. The MIC for this conjugate was 8 μg/mL, which was higher than that of the monoconjugate. Therefore, DFOA not complexed with Fe^3+^ does not work as a vector for targeted delivery. The antimicrobial activity results obtained with conjugates with DFOA in the presence and absence of Fe^3+^ ions are in agreement with the data reported in other studies [37,44].

Finally, the examination of a series of PMAG(10)-(DFOA,CT-B12) complex conjugates with aldimine or reduced aldimine bonds and in the presence or absence of Fe^3+^ demonstrated the same tendency (Figure 5c). Specifically, the lowest MIC (16 μg/mL) was observed for the PMAG(10)-(DFOA+Fe,CT-B12) conjugate with aldimine bonds and containing complexed iron ions. Recently, Shahzad et al. reported that the combination of some vitamins with antibiotics can provide a synergistic antimicrobial effect [64]. In particular, vitamin B12 was found to be effective in combination with PMX B. However, the authors used dissolved free forms of the components. In our case, all conjugates containing vitamin B12 showed higher MIC values than conjugates of similar composition but without B12. This can be explained by the fact that vitamin B12 was conjugated to CT and that the conjugation efficacy was close to 100%. This means that almost every molecule of CT contained conjugated vitamin B12. Considering the large size of vitamin B12, it may create steric hindrances when interacting with bacteria. Furthermore, the conjugation of large amounts of vitamin B12 affected the consumption of some amino groups of CT, which, in turn, may affect the activity of the antibiotic.

#### 3.2.2. Cytotoxicity

**Figure 6:**
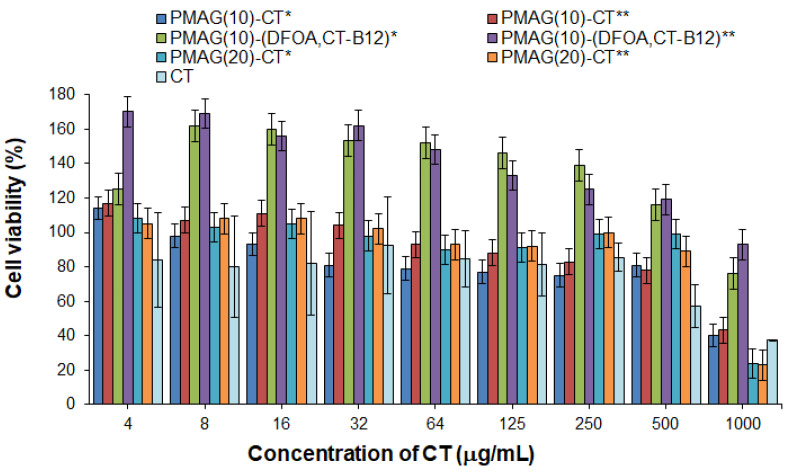
Cytotoxicity of CT and its various PMAG-based conjugates in HEK 293 cells (MTT, 72 h). * Non-reduced aldimine bonds; ** reduced aldimine bonds.

Polymyxins are known to possess nephro- and neurotoxicity [9,65]. In this regard, the cytotoxicity of free CT and its conjugates was evaluated and compared using human embryonic kidney cells (HEK 293, MTT, 72 h). The absence of toxicity for free CT was found up to a concentration of 250 μg/mL, whereas at a concentration of 500 μg/mL, moderate cytotoxicity was detected (the viability of HEK 293 was about 60%) (Figure 6). In turn, the absence of cytotoxicity at a concentration of 500 μg/mL was observed for all conjugates examined in the study. The same trend was previously reported by Dubashinskaya et al. for polymyxin E conjugates with hyaluronic acid [33].

At the same time, vitamin B12-containing complex conjugates were nontoxic over the entire concentration range (up to 1000 μg/mL) (Figure 6). In addition, these conjugates showed marked proliferative activity at concentrations up to 500 μg/mL for HEK 293 cells. The ability of vitamin B12 to stimulate cell proliferation is a known property of this substance [66].

#### 3.2.3. Caco-2 Cell Permeability

For B12-containing complex conjugates with non-reduced and reduced aldimine bonds, namely, PMAG(10)-(DFOA,CT-B12)* and PMAG(10)-(DFOA,CT-B12)**, their intestinal absorption was evaluated in comparison with free colistin and vitamin B12. In this experiment, CT, which is known to have low absorption in the intestine [67], was used as a negative control. In turn, vitamin B12 has a very high permeability in the intestine [68], so it was used as a positive control.

Determination of the apparent permeability coefficients (*P_app_*) across Caco-2 monolayers grown on semipermeable substrate is a fairly simple and widely used technique for predicting the absorption of the drugs and drug delivery systems developed for oral delivery [69]. Although the Caco-2 cell line is cancerous and derived from colon carcinoma, one of the key properties of Caco-2 cells is their ability to differentiate into a monolayer of cells with properties characteristic of absorptive intestinal enterocytes [70]. Specifically, Caco-2 monolayers can form a brush border on the apical surface, tight junctions, and microvilli as in the small intestine [71].

**Figure 7:**
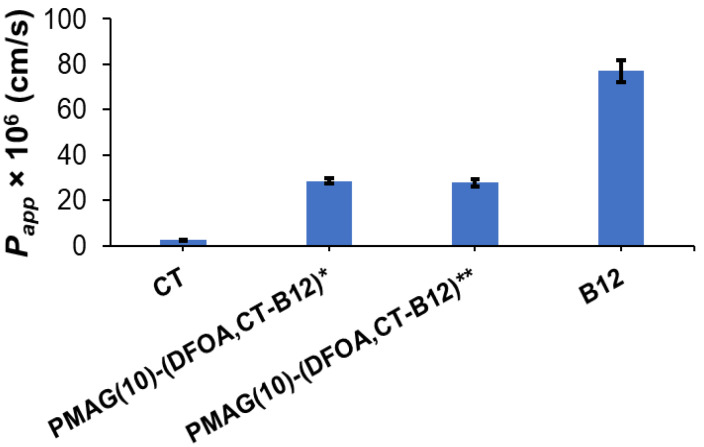
Apparent permeability coefficients (*P_app_*) for vitamin B12 (positive control), PMAG(10)-(DFOA, CT-B12) conjugates, and CT (negative control) for 2 h. * Non-reduced aldimine bonds; ** reduced aldimine bonds.

The results regarding the apparent permeability of the conjugates and controls are shown in Figure 7. As expected, free CT showed a very low permeability coefficient (*P_app_* ~3 × 10^−6^ cm/s), while vitamin B12 effectively crossed the Caco-2 monolayer (*P_app_* ~77 × 10^−6^ cm/s). The permeability of both tested complex CT-containing conjugates (*P_app_* 28–29 × 10^−6^ cm/s) was higher than for free CT. A similar improvement in permeability compared to free CT has been recently reported for the conjugate of CT and vitamin B12 with hyaluronic acid [51].

### 3.3. Stability of Conjugates in Gastrointestinal Simulated Media

Systems intended for oral delivery must meet stability criteria in gastrointestinal conditions before they are absorbed into the small intestine. It is known that the fasted stomach and intestine have pH 1–2 and 6.5–7.0, respectively [72]. In this study, the stability of complex conjugates under gastrointestinal conditions was investigated in media that simulate different parts of the gastrointestinal tract. For this purpose, hydrochloric acid solution (pH 1.0) and phosphate buffer (PB, pH 6.8) were used to resemble the pH of the fasted stomach and intestine fluids, respectively. In addition, biorelevant media simulating the properties and composition of gastrointestinal fluids, namely, FaSSGF and FaSSIF, were used to determine the stability of the conjugates. The average residence time in the stomach and intestine in the fasted state is usually about 2 h [73]. Therefore, this time was selected for the evaluation of the stability of the complex conjugates.

The stability results of PMAG(10)-(DFOA,CT) conjugates with hydrolyzable (aldimine) bonds and reduced aldimine bonds in different media are summarized in Table 4. It was shown that the release in the buffer and the simulated biorelevant medium was consistent. As expected, conjugates in which CT was linked to PMAG by aldimine bonds showed lower stability, resulting in a faster release of CT. Under gastric conditions, the release after 2 h was 20–26%, whereas under intestinal conditions, a release of 6–12% was detected. In turn, the reduced PMAG(10)-(DFOA,CT) conjugate exhibited a release of about 2% CT under intestinal conditions and 6–9% under gastric conditions. As for free CT, it is known that CT is more stable at acidic conditions than at pH 7.4 [74]. At pH 7.4 and 37 °C, the half-life of CT is 70 h.

### 3.4. Colistin Release from Conjugates

The in vitro release of CT from conjugates with aldimine (PMAG(10)-(DFOA,CT)*) and reduced aldimine (PMAG(10)-(DFOA,CT)**) bonds was studied for 48 h in two buffer solutions with pH 7.4 and 5.2 simulating the release in the blood and ileum (where active vitamin B12 absorption occurs [75]), and at the inflammatory site [76], respectively. In general, the release rate depends on the type of bonds connecting the drug in the polymer conjugate and their sensitivity to hydrolysis. For example, Dubashinskaya et al. showed that the release of CT from a conjugate with hyaluronic acid linked by amide bonds did not exceed 3 and 5% after a 24 h incubation in buffer solutions with pH 7.4 and 5.2, respectively [33]. A similar result was also shown for a polypeptide-based conjugate of polymyxin B (PMX B) [37] in which the drug was also linked to the polymer via amide bonds. In that case, the PMX B release was around 10% at pH 5.8, while it was even lower at pH 7.4.

**Figure 8:**
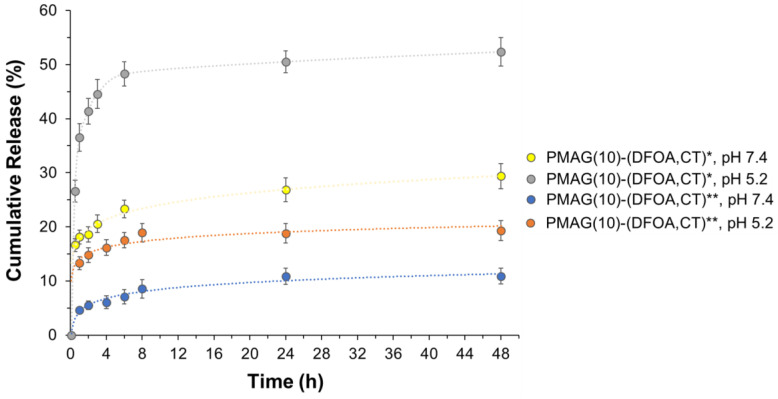
Release of CT from PMAG-based conjugates in 0.01 M PBS (pH 7.4) and citrate-phosphate buffer (pH 5.2) at 37 °C. * Non-reduced aldimine bonds; ** reduced aldimine bonds.

In the case of the synthesized PMAG-based conjugates, the most pronounced release was observed for the conjugate containing aldimine bonds (PMAG(10)-(DFOA,CT)*) most rapidly hydrolyzed at acidic conditions (Figure 8). For this conjugate, more than 50% of the CT was released after 48 h at pH 5.2. The release of CT from the same conjugate but at pH 7.4 was about 30%.

Although PMAG(10)-(DFOA,CT) containing aldimine bonds (Schiff base) was reduced with sodium borohydride (PMAG(10)-(DFOA,CT)**), release from this conjugate was detected. However, the amounts of CT released were ~18 and 10% at pH 5.2 and 7.4, respectively. As can be seen in Figure 2, in addition to aldimine (Schiff base) bonds, the product formed after oxidation of PMAG also contains labile hemiacetal bonds, which, like the Schiff bases, are efficiently cleaved under acidic conditions. Some hemiacetals may undergo hydrolysis during time-consuming purification prior to conjugation, providing the same aldehyde functionality that may be involved in the formation of aldimine bonds during conjugation. Both the Schiff bases and the remaining hemiacetal groups are sensitive to reduction by sodium borohydride and can be reduced by this agent [77,78]. According to literature data, the yields of secondary amines obtained by the reduction of Schiff bases using NaBH_4_ are in the range of 91–99% in small molecules and depend on the structure of linked radicals. The reduction of hemiacetals in small molecules can range from 6 to 85% depending on the conditions and structure of the compounds [79,80]. Considering that in our case, the polymeric products are reduced, and given that conversion in the polymer in analogous reactions does not reach 100%, it can be assumed that the reduction may not reach the total conversion of labile bonds. In addition, the purity of the obtained conjugates is around 96–98%. Thus, the detected release of CT from the reduced conjugate may be due to both the release of unbound CT (2–4%) and the incomplete reduction of labile bonds (6–16%). Given that both the aldimine and hemiacetal bonds are sensitive to acidic conditions, the increased release at pH 5.8 indirectly indicates the presence of a fraction of unreduced bonds.

## 4. Conclusions

A series of colistin conjugates based on a biocompatible synthetic glycopolymer, namely PMAG, was synthesized and thoroughly characterized by ^1^H NMR and FTIR spectroscopy, HPLC, and UV–Vis spectrometry, and the compositions of all conjugates were established. In addition to the monoconjugates, polymeric CT conjugates also containing DFOA (siderophore) as a vector for targeted delivery and vitamin B12, improving intestinal permeability, were obtained. All CT conjugates showed higher biocompatibility with normal cells (HEK 293) than the free antibiotic. Moreover, the presence of vitamin B12 in the conjugate enhanced cell proliferation. Conjugates in which the components were linked to the polymer via aldimine bonds demonstrated a faster drug release rate, while conjugates with reduced aldimine bonds were characterized by slower release and increased stability in the simulated gastrointestinal fluids. As expected, vitamin B12-containing conjugates showed improved permeability through the Caco-2 model monolayer simulating intestinal enterocytes compared to free CT. All CT conjugates possessed antibacterial activity. The best antibacterial activity was detected for complex conjugates containing DFOA in complexes with Fe^3+^ ions. Conjugation of vitamin B12 in almost every CT molecule contributed to a certain increase in the MIC values. Presumably, the conjugation of a lower amount of vitamin B12 to CT may preserve its activity to a greater extent.

In summary, PMAG-(DFOA+Fe,CT) conjugates can be considered as promising targeted systems for intravenous administration, while PMAG-(DFOA+Fe,CT-B12) conjugates are suitable for oral delivery.

## Figures and Tables

**Table 1:** Efficacy of CT and DFOA conjugation to oxidized PMAG depending on the initial content of aldehyde groups in PMAG.

Conjugate *^a^*	Content of CHO-Groups in PMAG (mol%)	Initial Amounts of Substances (*m*_0_) *^b^* (μg/mg PMAG)		Content of Conjugated Substances (*m_C_*) (μg/mg PMAG)		Conjugation Efficacy *^c^* (%)	
		CT	DFOA	CT	DFOA	CT	DFOA *^d^*
PMAG(10)-CT	10	870	-	454 ± 39	-	52 ± 4	-
PMAG(20)-CT	20	1740	-	710 ± 81	-	41 ± 5	-
PMAG(10)-DFOA	10	-	2680	-	218 ± 22	-	41 ± 4
PMAG(20)-DFOA	20	-	5360	-	610 ± 54	-	57 ± 5
PMAG(10)-(DFOA,CT)	10	870	2680	415 ± 36	294 ± 31	48 ± 4	55 ± 6
PMAG(20)-(DFOA,CT)	20	1740	5360	791 ± 97	563 ± 47	45 ± 6	53 ± 4

*^a^* The purity of conjugates was in the range of 96–98%. *^b^* The molar ratio of aldehyde groups:CT:DFOA was 1:1:5. *^c^* Calculated using Equation (1) (see Section 2.2.2). *^d^* For calculation of CE (%) in the case of DFOA, *m*_0_ = *m*/5.

**Table 2:** Efficacy of Suc-B12 conjugation with PMAG(10)-(DFOA,CT) conjugates.

Conjugate *^a^*	Initial Amount of Suc-B12 (B12) *^b^* (μg/mg Conjugate)	Amount of Conjugated Suc-B12 (B12) (μg/mg Conjugate)	Conjugation Efficacy (%)
PMAG(10)-(DFOA,CT-B12) (*aldimine bonds*)	363 (339)	356 ± 7 (332 ± 7)	98 ± 2
PMAG(10)-(DFOA,CT-B12) (*reduced aldimine bonds*)	363 (339)	352 ± 11 (329 ± 10)	97 ± 3

*^a^* The purity of conjugates was 98%. *^b^* The molar ratio of CT:Suc-B12 was 1:1.

**Table 3:** Composition of the conjugates used in the study and the contents of individual components per mg of the conjugate. Each conjugate was obtained in two forms, namely, with non-reduced and reduced aldimine bonds.

Conjugate	Content of Components in Conjugate (μg/mg Conjugate)		
	CT	DFOA	B12
PMAG(10)-CT	312	–	–
PMAG(20)-CT	415	–	–
PMAG(10)-DFOA	–	179	–
PMAG(20)-DFOA	–	379	–
PMAG(10)-(DFOA,CT)	243	172	–
PMAG(20)-(DFOA,CT)	336	239	–
PMAG(10)-(DFOA,CT-B12)	180	127	244

**Table 4:** PMAG(10)-(DFOA, CT) stability under conditions simulating various gastrointestinal areas at 37 °C.

Conjugate	Conditions	Time (h)	Release of CT (%)
PMAG(10)-(DFOA, CT) *	FaSSGF (pH 1.6)	2	20 ± 4
	FaSSIF (pH 6.5)	2	6 ± 2
	0.1 M HCl (pH 1.0)	2	26 ± 6
	PB (pH 6.8)	2	9 ± 2
PMAG(10)-(DFOA, CT) **	FaSSGF (pH 1.6)	2	6 ± 2
	FaSSIF (pH 6.5)	2	~2
	0.1 M HCl (pH 1.0)	2	12 ± 2
	PB (pH 6.8)	2	~2

* Non-reduced aldimine bonds; ** reduced aldimine bonds.

## Data Availability

All data are available within the article and its Supplementary Materials.

## Supplementary Materials

The following information can be downloaded at: https://www.mdpi.com/article/10.3390/pharmaceutics16081080/s1, Figure S1: ^1^H NMR spectra (400 MHz, D_2_O, 25 °C): (a) PMAG, (b) colistin, (c) deferoxamine, (d) cyanocobalamin (vitamin B12), (e) PMAG(10)-(DFOA,CT) complex conjugate with reduced aldimine bonds (1D-DOSY ^1^H NMR spectrum), (f) complex PMAG(10)-(DFOA,CT,B12) conjugate with reduced aldimine bonds (1D-DOSY ^1^H NMR spectrum); Figure S2: Images of PMAG (left, white) and conjugates with reduced aldimine bonds, namely, PMAG(10)-(DFOA,CT) (middle, yellow) and PMAG(10)-(DFOA,CT,B12) (right, crimson red). Figure S3. FTIR spectra of PMAG-based conjugates and neat substances. Ref. [81] is cited in the Supplementary Materials.
