# Supplementary material for: Poly(2-Deoxy-2-Methacrylamido-D-Glucose)-Based Complex Conjugates of Colistin, Deferoxamine and Vitamin B12: Synthesis and Biological Evaluation
Source: Pharmaceutics. 2024 Aug 17;16(8):1080. doi: 10.3390/pharmaceutics16081080 (PMC11359296; doi:10.3390/pharmaceutics16081080)
Supplement: Supplementary file 1 [file pharmaceutics-16-01080-s001.zip › pharmaceutics-3138326-supplementary.pdf]

pharmaceutics

Supplementary Materials: Poly(2-Deoxy-2-Methacrylamido-D-Glucose)-Based Complex Conjugates of Colistin, Deferoxamine and Vitamin B12: Synthesis and Biological Evaluation

Mariia Stepanova, Mariia Levit, Tatiana Egorova, Yulia Nashchekina, Tatiana Sall, Elena Demyanova, Ivan Guryanov and Evgenia Korzhikova-Vlakh

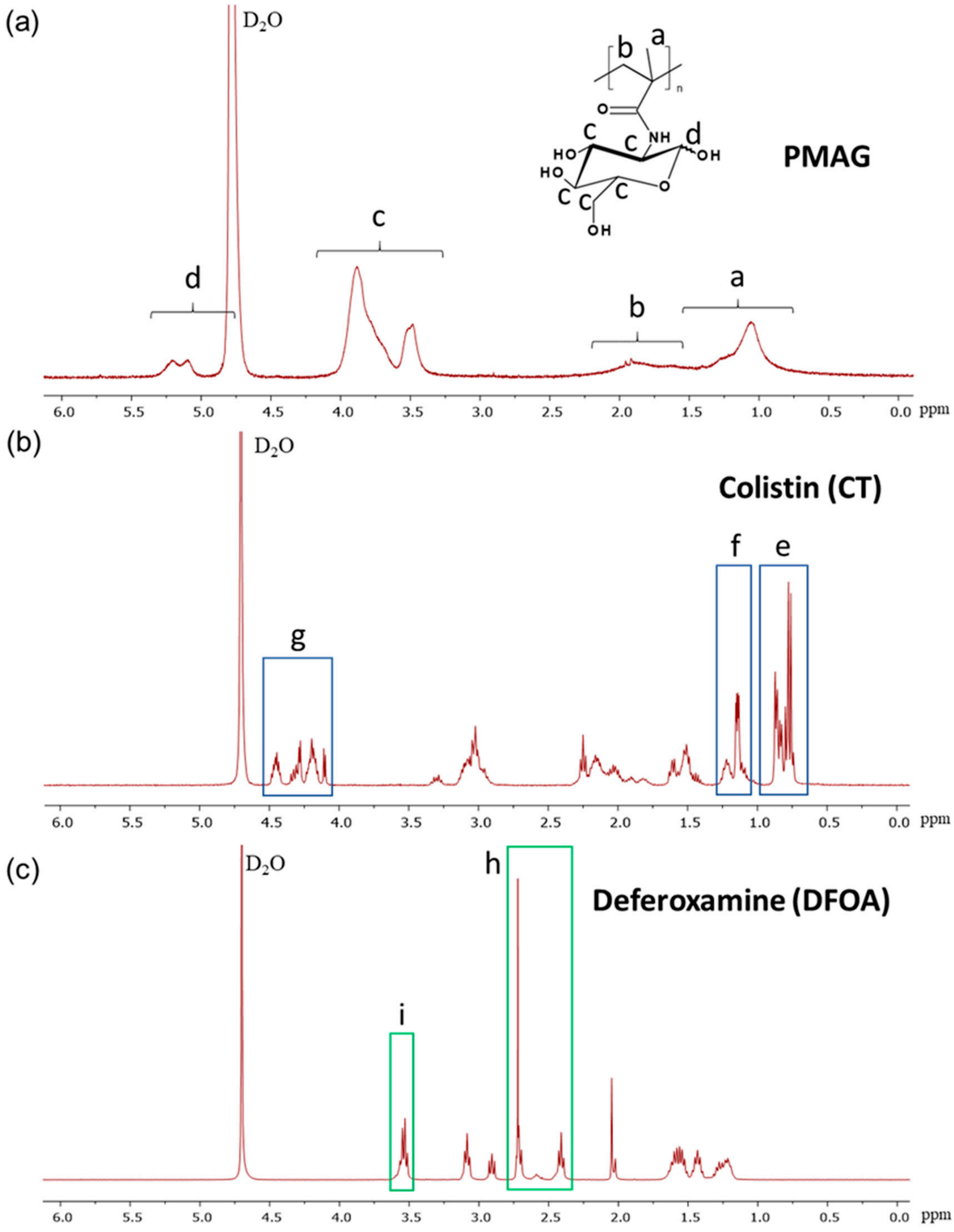

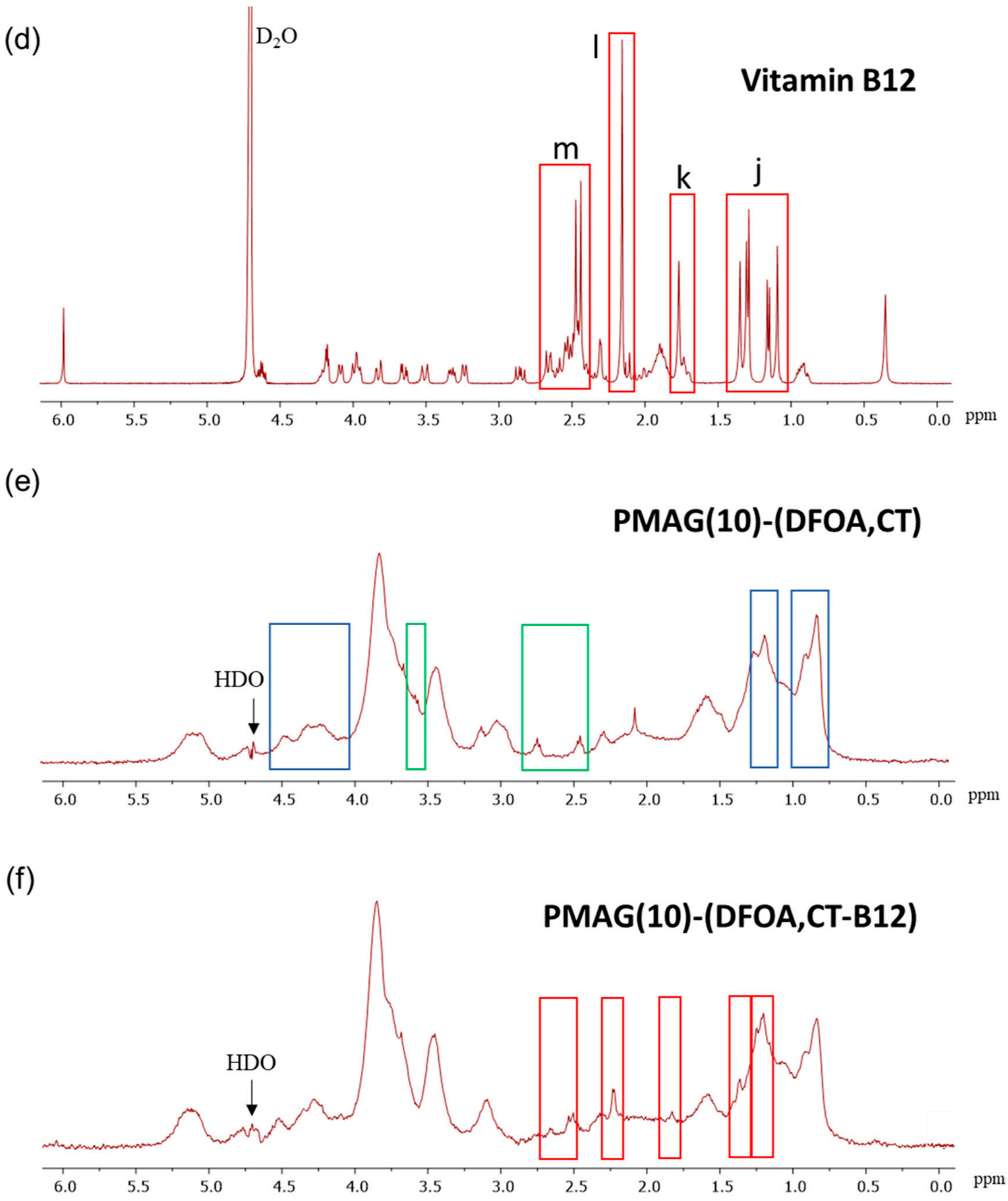

Figure S1. ^{1}H NMR spectra (400 MHz, D_2O, 25 °C): (**a**) PMAG, (**b**) colistin, (**c**) deferoxamine, (**d**) cyancobalamine (vitamin B12), (**e**) PMAG(10)-(DFOA,CT) complex conjugate with reduced aldimine bonds (1D-DOSY ^{1}H NMR spectrum), (**f**) complex PMAG(10)-(DFOA,CT,B12) conjugate with reduced aldimine bonds (1D-DOSY ^{1}H NMR spectrum).

Highlighted signals: **a** and **b** – $-CH_3$ and $-CH_2-$ groups of the PMAG backbone, respectively, and **c** and **d** – -CH- and $-CH_2-$ groups of PMAG glucose ring (see Ref. [55] in the main text); **e** and **f** – a series of $-CH_3$, $-CH_2-$ and -CH- groups of 6-methylheptanoic acid, leucine and threonine residues of CT (see Refs. [57] and [58] in the main text); **g** – -$C^{\alpha}H$ of threonine, leucine and 2,4-diaminobutiric acid and -$C^{\beta}H$ of threonine residues of CT (Ref. [58] in the main text); **h** and **i** – $-CH_2C(O)-$ and -C<u>H_2</u>-NH- groups of DFOA (Ref. [59] in the main text), respectively; **j** and **m** – $-CH_3$ and $-CH_2-$ groups, and **k** and **l** – only $-CH_2-$ groups of various substituents of the B12 core ring (Ref. [81] in the main text).

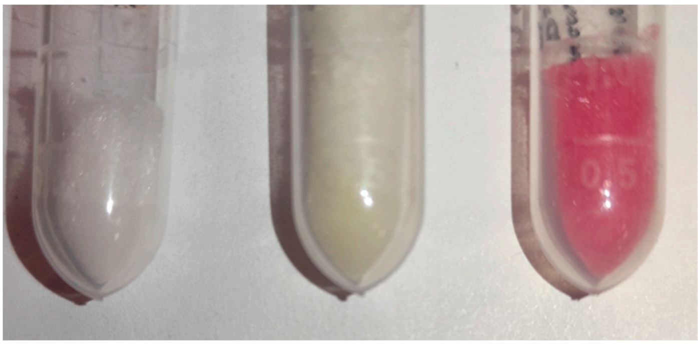

Figure S2. Images of PMAG (left, white) and conjugates with reduced aldimine bonds, namely PMAG(10)-(DFOA,CT) (middle, yellow) and PMAG(10)-(DFOA,CT,B12) (right, crimson red).

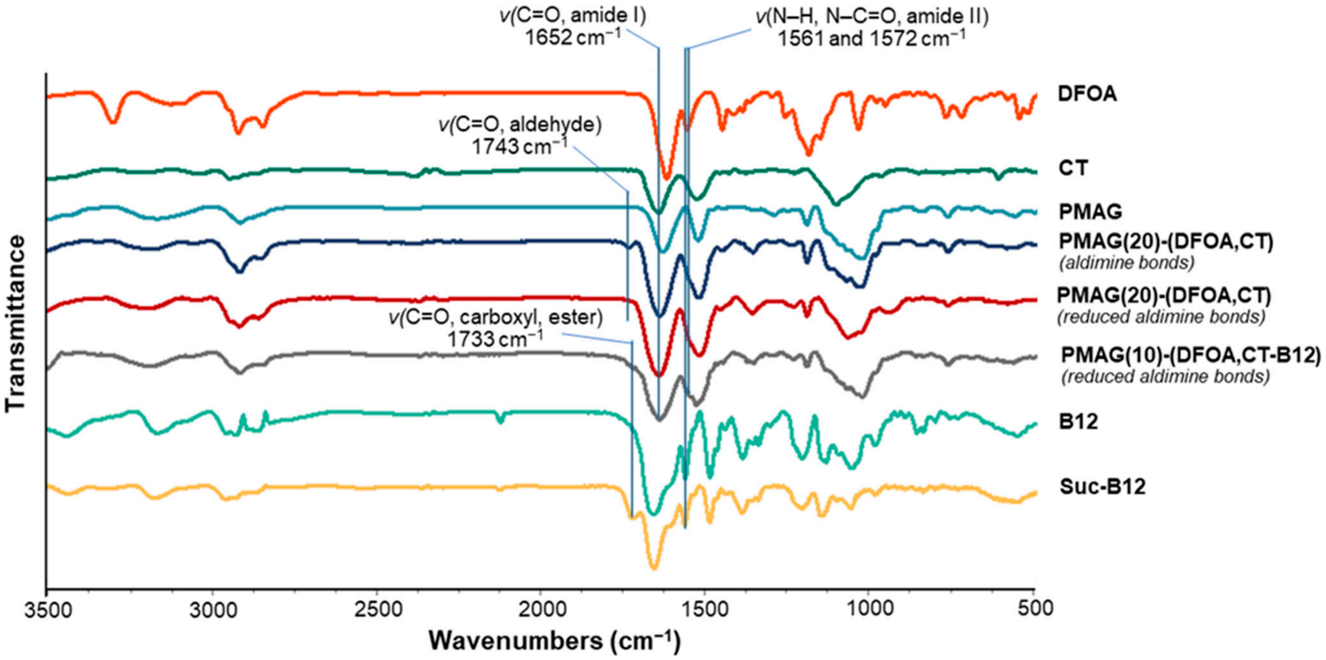

Figure S3. FTIR spectra of PMAG-based conjugates and neat substances.
